# Supplementary material for: Evidence supported by Mendelian randomization: impact on inflammatory factors in knee osteoarthritis
Source: Front Med (Lausanne). 2024 May 28;11:1382836. doi: 10.3389/fmed.2024.1382836 (PMC11165061; doi:10.3389/fmed.2024.1382836)
Supplement: Supplementary file 1 [file Data_Sheet_1.ZIP › data/GCST90274801_5e6/801.forest.pdf]

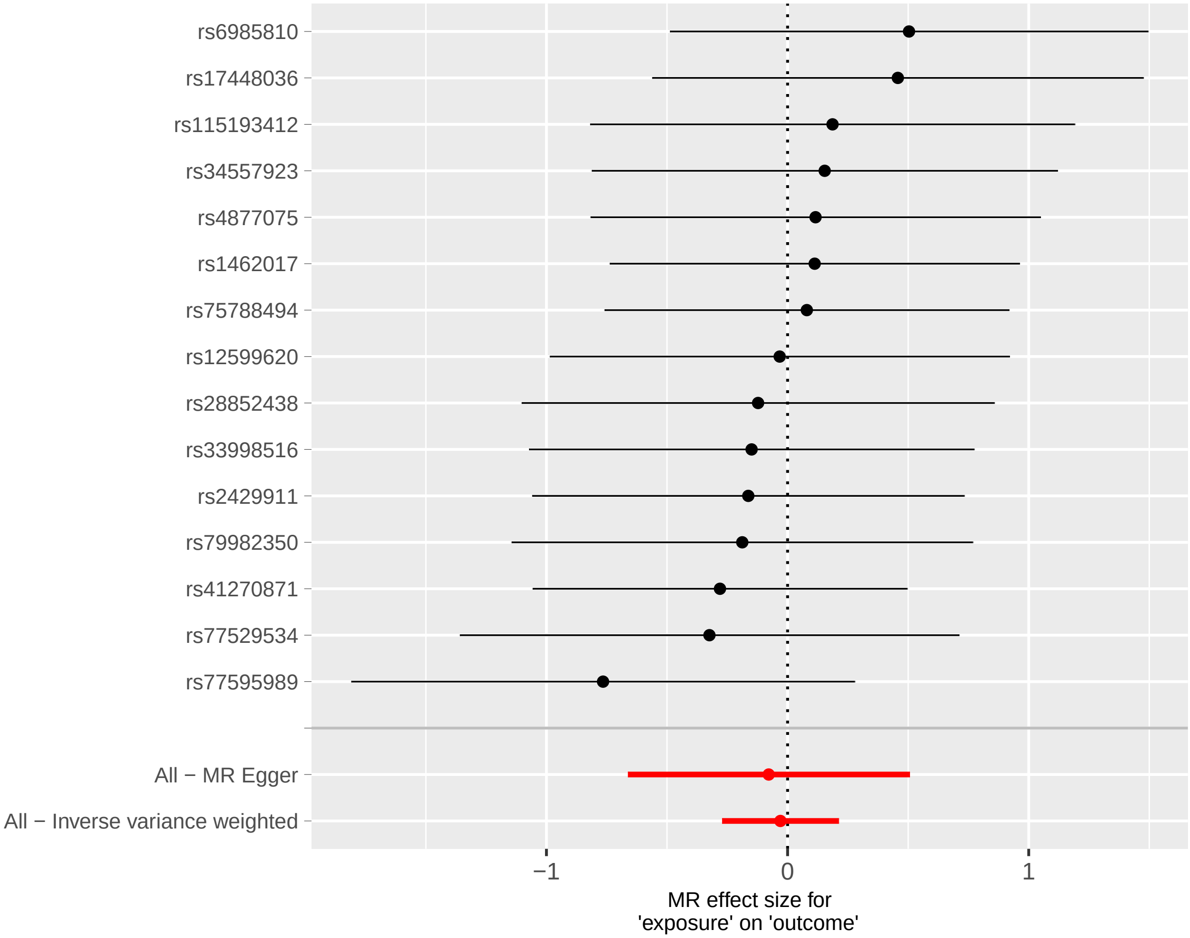

rs6985810
rs17448036
rs115193412
rs34557923
rs4877075
rs1462017
rs75788494
rs12599620
rs28852438
rs33998516
rs2429911
rs79982350
rs41270871
rs77529534
rs77595989
All − MR Egger
All − Inverse variance weighted
−1
0
1
MR effect size for
'exposure' on 'outcome'
